# Supplementary material for: SirA inhibits the essential DnaA:DnaD interaction to block helicase recruitment during Bacillus subtilis sporulation
Source: Nucleic Acids Res. 2022 Nov 23;51(9):4302–21. doi: 10.1093/nar/gkac1060 (PMC10201431; doi:10.1093/nar/gkac1060)
Supplement: gkac1060_Supplemental_Files [file gkac1060_supplemental_files.zip › SirA-DnaD_figures_SUPPLEMENTARY.pdf]

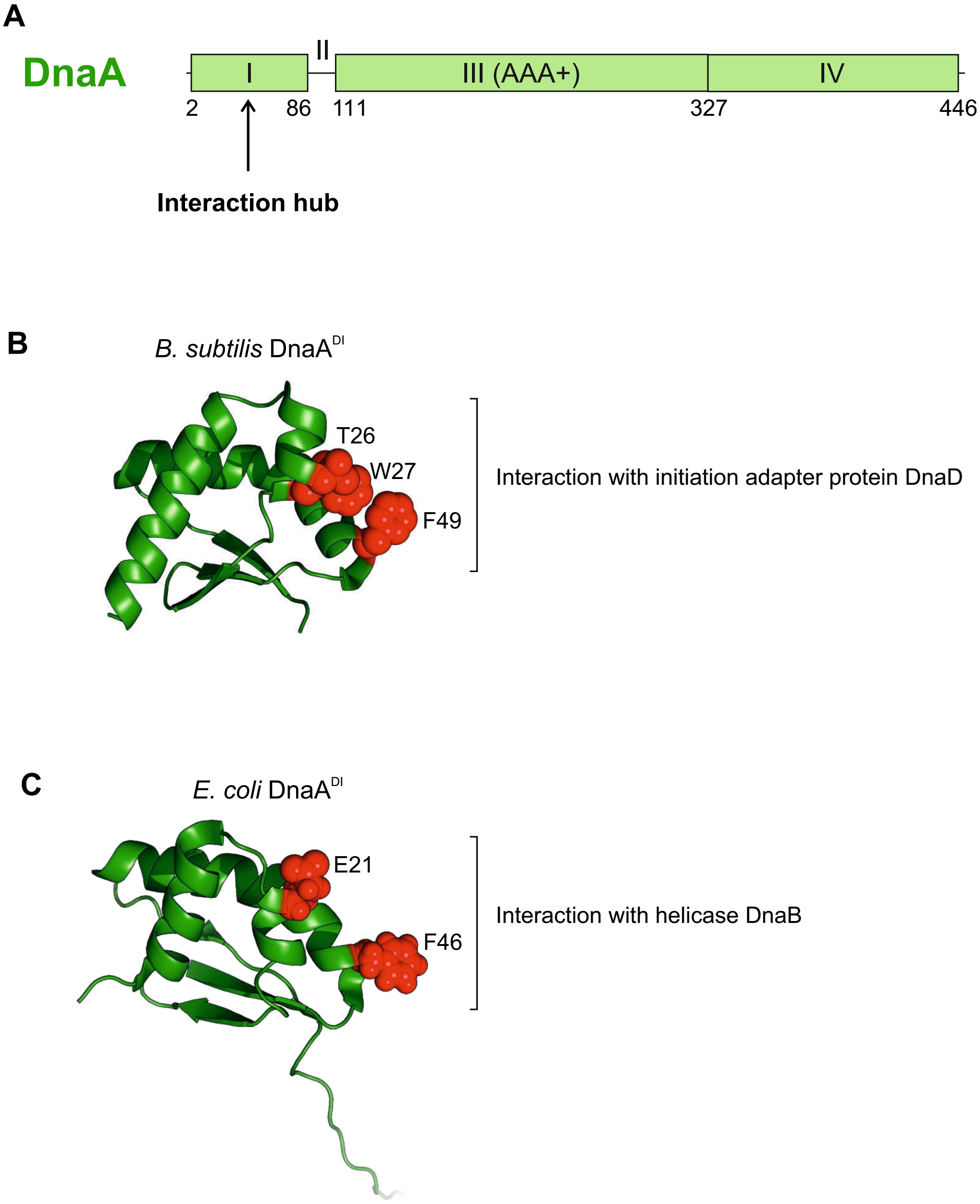

Figure S1. Domain organisation of DnaA highlighting a shared interaction hub in domain I. (A) *B. subtilis* DnaA domain organisation with amino acid boundaries indicated. **(B)** Crystal structure of *B. subtilis* DnaA domain I (PDB 4TPS) with residues thought to be involved in protein-protein interactions highlighted in red. **(C)** NMR structure of *E. coli* DnaA domain I (PDB 2E0G) with residues thought to be involved in protein-protein interactions highlighted in red.

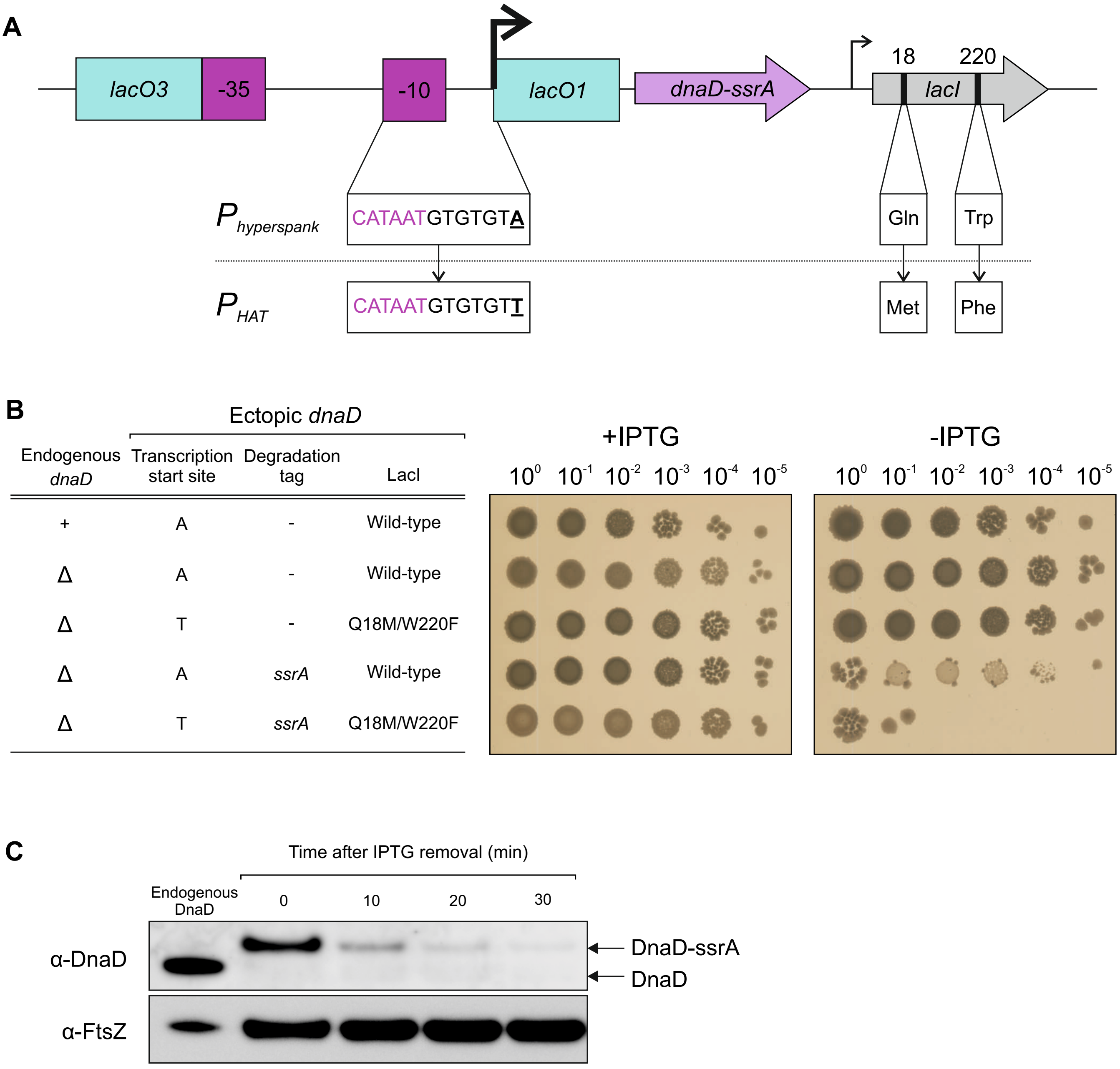

Figure S2. Construction of the inducible *dnaD-ssrA* strain. (A) Schematics of the inducible system used to drive the expression of the *dnaD-ssrA* fusion. The transcription start site (+1) of the $P_{hyperspank}$ promoter was mutated from A to T (P_{HAT}) and the *lacI* repressor binding to operator sites lacO1/lacO3 was tightened by the combination of mutations $LacI^{Q18M/W220F}$. **(B)** Spot-titre assay showing the combination required to achieve conditional DnaD-SsrA complementation. The presence or absence of IPTG indicates that the ectopic *dnaD* copy is turned on or off, respectively. From top to bottom, strains are CW2, CW231, CW103, CW232 and CW164. **(C)** Immunoblot analysis of the inducible *dnaD-ssrA* cassette showing that degradation of DnaD-SsrA is achieved in about 30 minutes post-depletion of IPTG (CW164); endogenous *dnaD* indicates expression of wild-type DnaD in *B. subtilis* 168CA. Detection of the tubulin homolog FtsZ was used as a loading control.

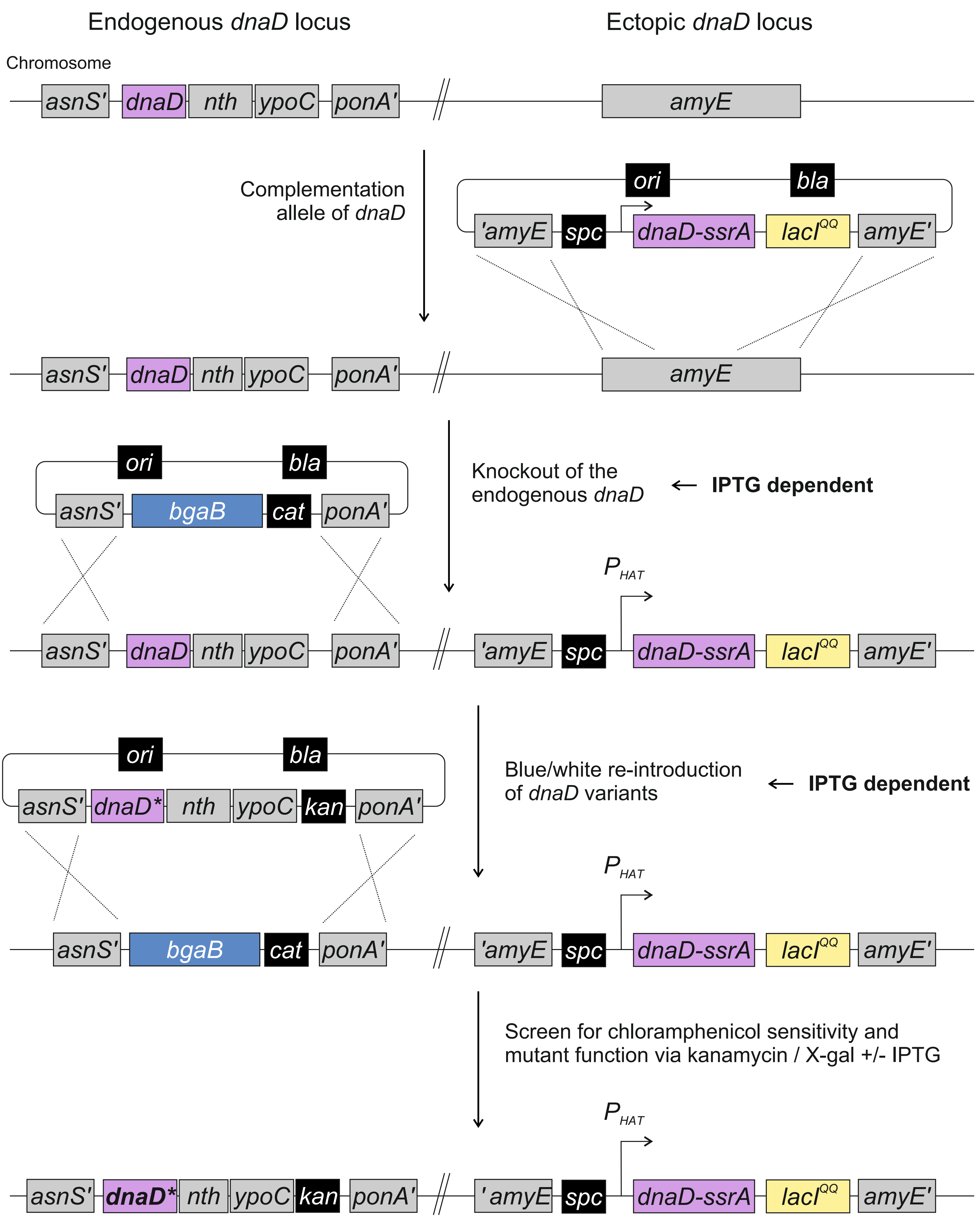

Figure S3. Methodology for genetic complementation and introduction of *dnaD* mutants. Schematics of the blue/white screening assay. In *B. subtilis* 168CA, the inducible complementation cassette *dnaD-ssrA* was inserted at the *amyE* locus (CW162), followed by replacement of the native *dnaD* operon by a *bgaB* cassette (encoding β-galactosidase, CW197). In the presence of the chromogenic substrate X-gal, colonies containing the *bgaB* cassette appear blue. Selection of *dnaD* mutants is performed in the presence of kanamycin, X-gal (blue/white) and IPTG (functional complementation). The function of individual mutants is then tested by growing cells in the presence or absence of IPTG.

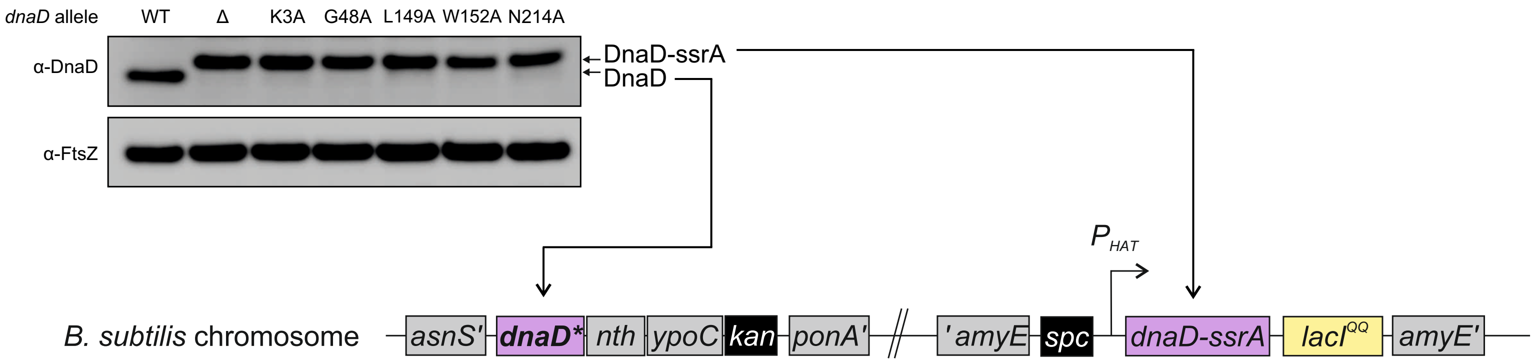

Figure S4. Essential DnaD residues that are not expressed *in vivo*. Immunobloting shows that some lethal alanine substitutions in the endogenous copy of DnaD were not well expressed *in vivo*. Only the expression of the ectopic DnaD-SsrA copy could be detected, whereas expression of the mutants was comparable to a strain lacking an endogenous copy of *dnaD*. Detection of the tubulin homolog FtsZ was used as a loading control. Wild-type (*B. subtilis* 168CA), Δ (CW197), K3A (CW289), G48A (CW293), L149A (CW308), W152A (CW302), N214A (CW283). Relevant schematics of the chromosome show which proteins are associated with the detected DnaD bands on the immunoblot.

A

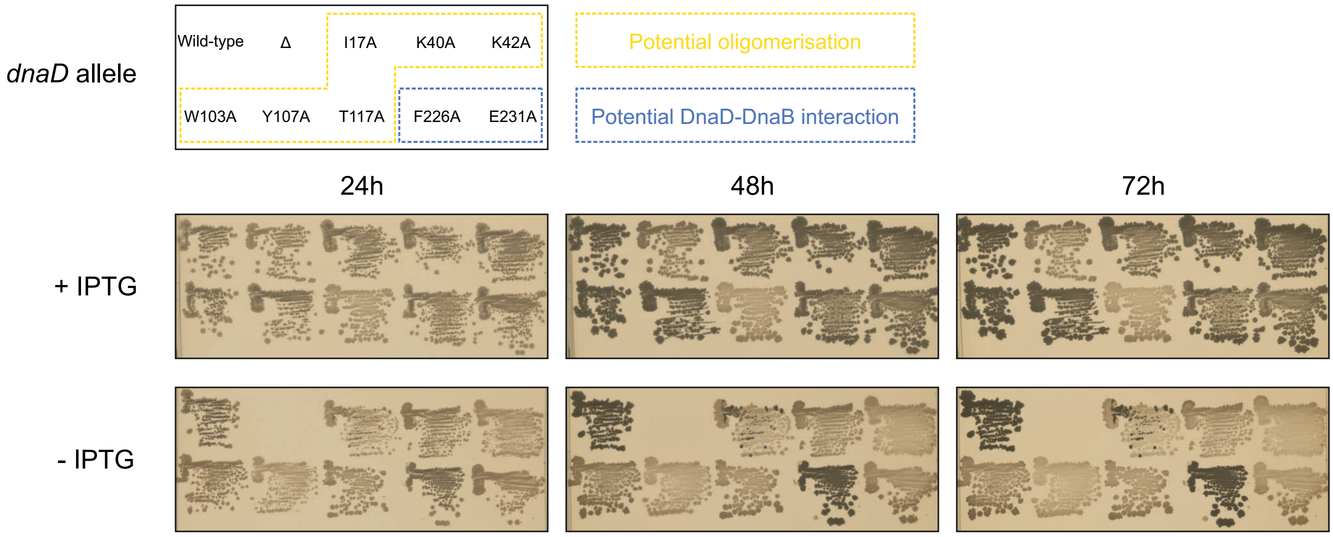

B

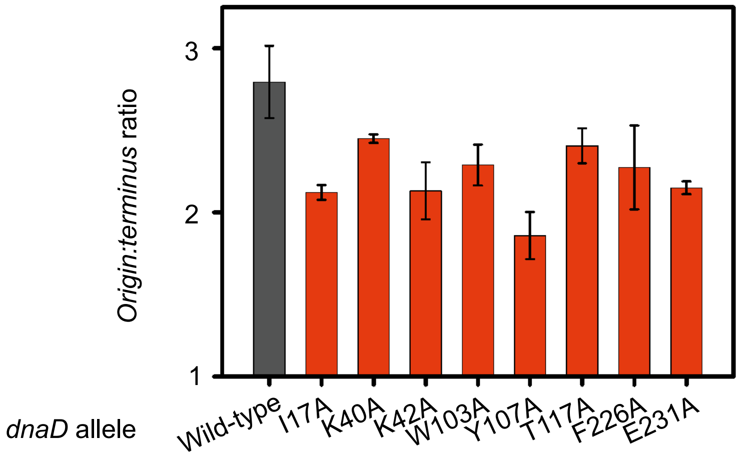

Figure S5. Analysis of DnaD intermediate phenotype mutants. (A) Colony restreaks of DnaD mutants that are associated with a translucent phenotype or leading to heterogeneous colonies over 72h growth at 37°C. Plates are shown in the presence (+IPTG) or absence (-IPTG) of *dnaD-ssrA* and their corresponding layout is detailed at the top (WT indicates wild-type). The location of these residues in DnaD structure suggests they are involved in oligomerisation or contribute to the DnaD-DnaB interaction. Wild-type (CW162), Δ (CW197), I17A (CW290), K40A (CW306), K42A (CW307), W103A (CW298), Y107A (CW299), T117A (CW300), F226A (CW284), E231A (CW309). **(B)** Marker frequency analysis by quantitative PCR associated with strains shown in panel (A) in the absence of DnaD-SsrA. Primers used to amplify the origin annealed within the *incC* region. Strains are the same as shown in (A).

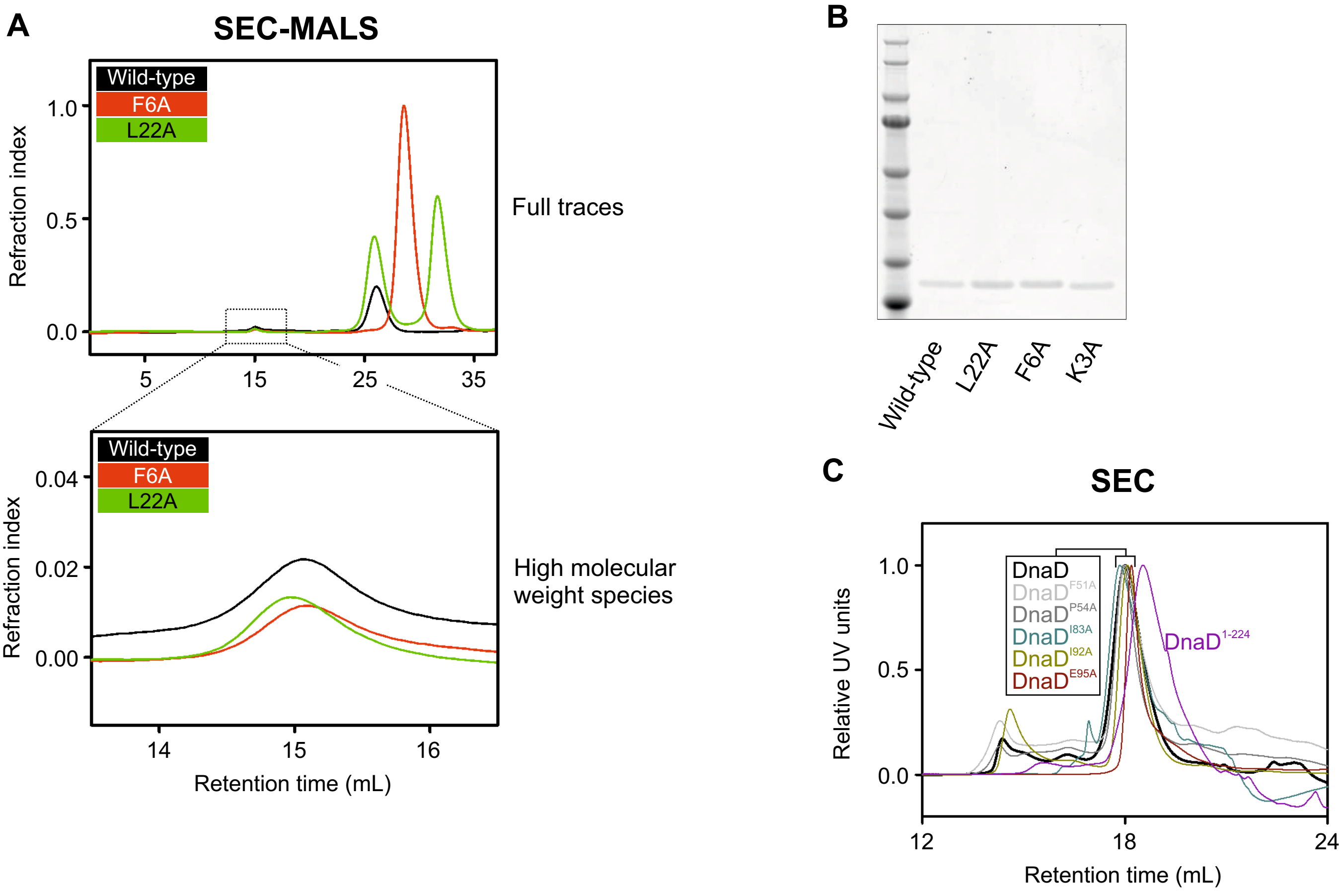

Figure S6. Size-exclusion chromatography and crosslinking analysis controls. (A) Full trace of the UV-spectrum represented as a refraction index during the SEC experiments showing that the DnaD variants (wild-type, F6A and L22A) did not display major fractions of aggregates (high molecular weight species). **(B)** Coomassie staining of the DnaD variants (wild-type, L22A, F6A and K3A) that were used during the BS3 crosslinking assay showing that all species were approximately used in equimolar amounts. **(C)** SEC profiles of other DnaD point mutants (DnaD^{F51A}, DnaD^{P54A}, DnaD^{I83A}, DnaD^{I92A}, DnaD^{E95A}) and a C-terminal truncation of the last eight DnaD amino acids (DnaD$^{1\text{-}224}$) showing that these variants retained a structure matching that of the wild-type protein.

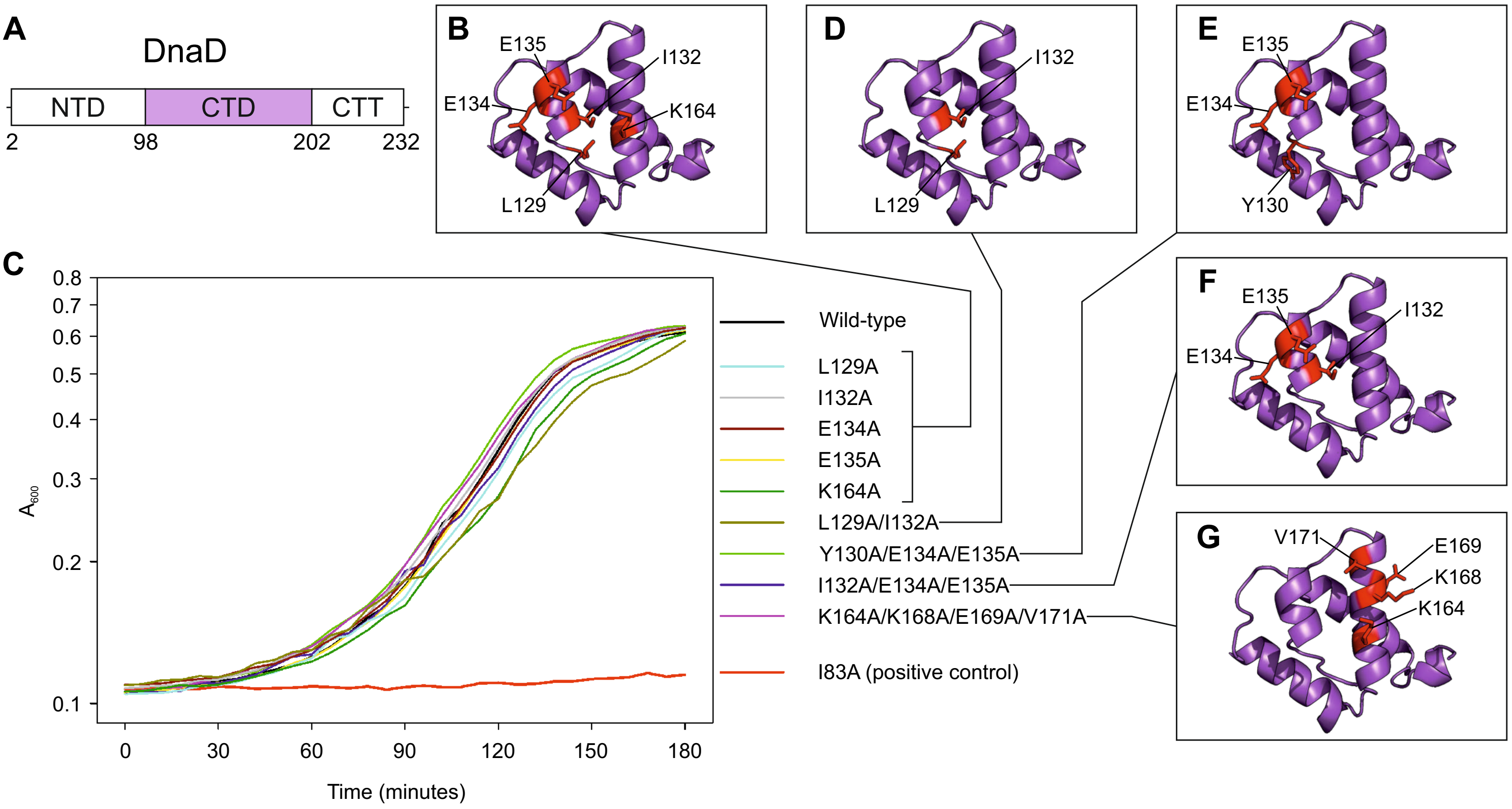

Figure S7. Residues in DnaD C-terminal domain that interact with DnaA^{DI} *in vitro* are not essential *in vivo*. (A) Domain organisation of DnaD with amino acid boundaries indicated. NTD denotes the N-Terminal Domain, CTD the C-Terminal Domain and CTT the C-Terminal Tail of DnaD. **(B)** Individual substitutions in DnaD^{CTD} mapped onto the NMR structure. **(C)** Growth analysis of *B. subtilis* DnaD variants using the inducible *dnaD-ssrA* strain in the absence of IPTG. Individual or combinations of mutations in DnaD^{CTD} show that growth was unaffected by these changes. Strains in legend (from top to bottom) are: CW162, CW179, CW167, CW171, CW172, CW173, CW176, CW177, CW178, CW168, CW170. **(D-G)** Multiple substitutions in DnaD C-terminal domain that were used in (C) mapped onto the NMR structure (37).

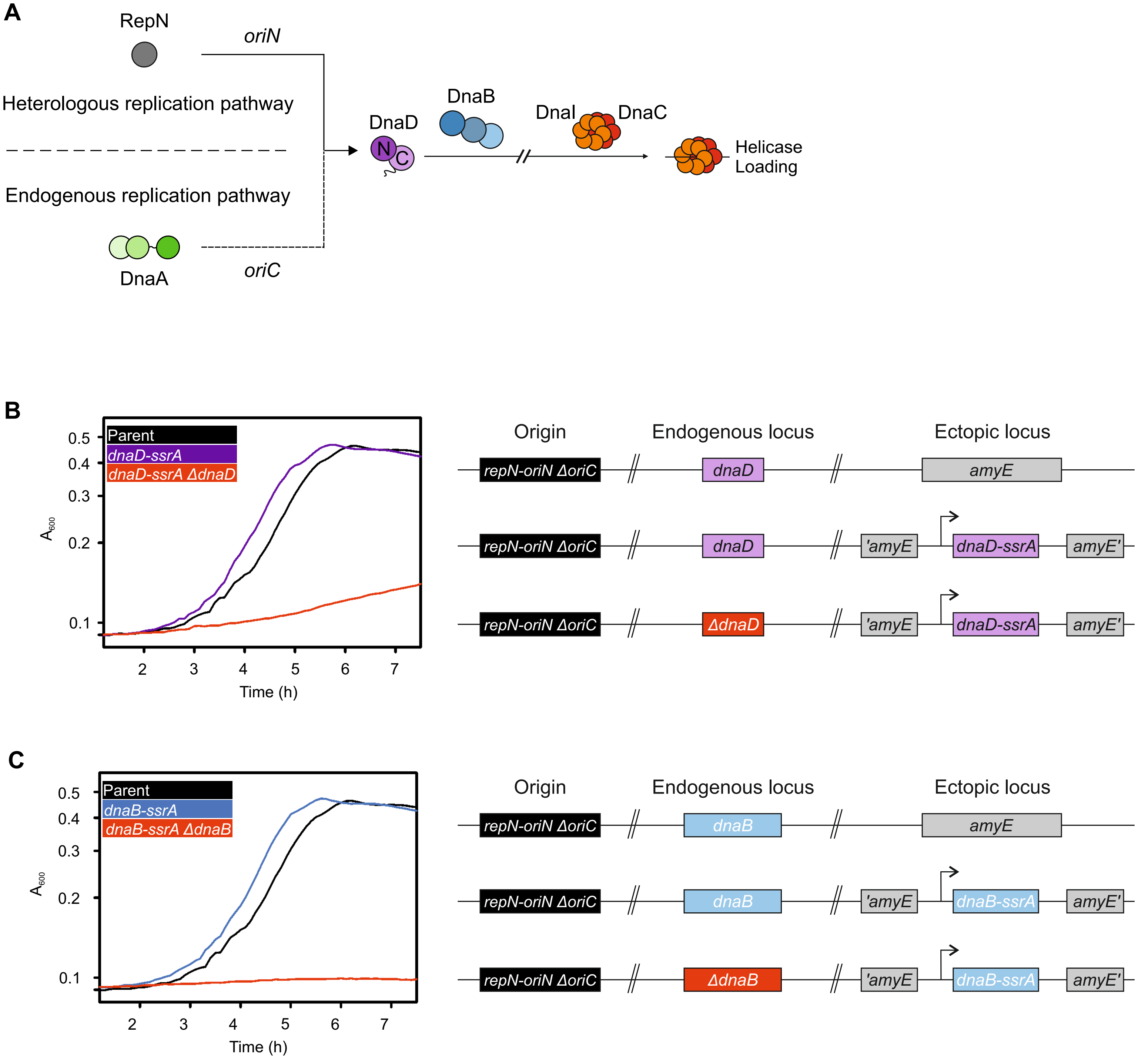

Figure S8. Endogenous and heterologous DNA replication systems used for the study of DnaA variants in *B. subtilis*. (A) Endogenous replication via DnaA at *oriC* can be complemented by the presence of the heterologous *oriN-repN* replication system. Here, this allows the study of DnaA or origin mutants without acquiring suppressor mutations. Note that both pathways require DnaD and DnaB to achieve helicase loading. **(B)** Plate reader assay showing growth of a strain replicating exclusively via *oriN*, with (*dnaD-ssrA*, CW651) and without (*dnaD-ssrA ΔdnaD*, CW658) DnaD expression. **(C)** Plate reader assay showing growth of a strain replicating exclusively via *oriN,* with (*dnaB-ssrA*, CW652) and without (*dnaB-ssrA ΔdnaB*, CW659) DnaB expression. Parent in (B-C) shows the growth profile of a strain replicating exclusively via *oriN* that only contains the native copies of *dnaD* and *dnaB* (HM950).

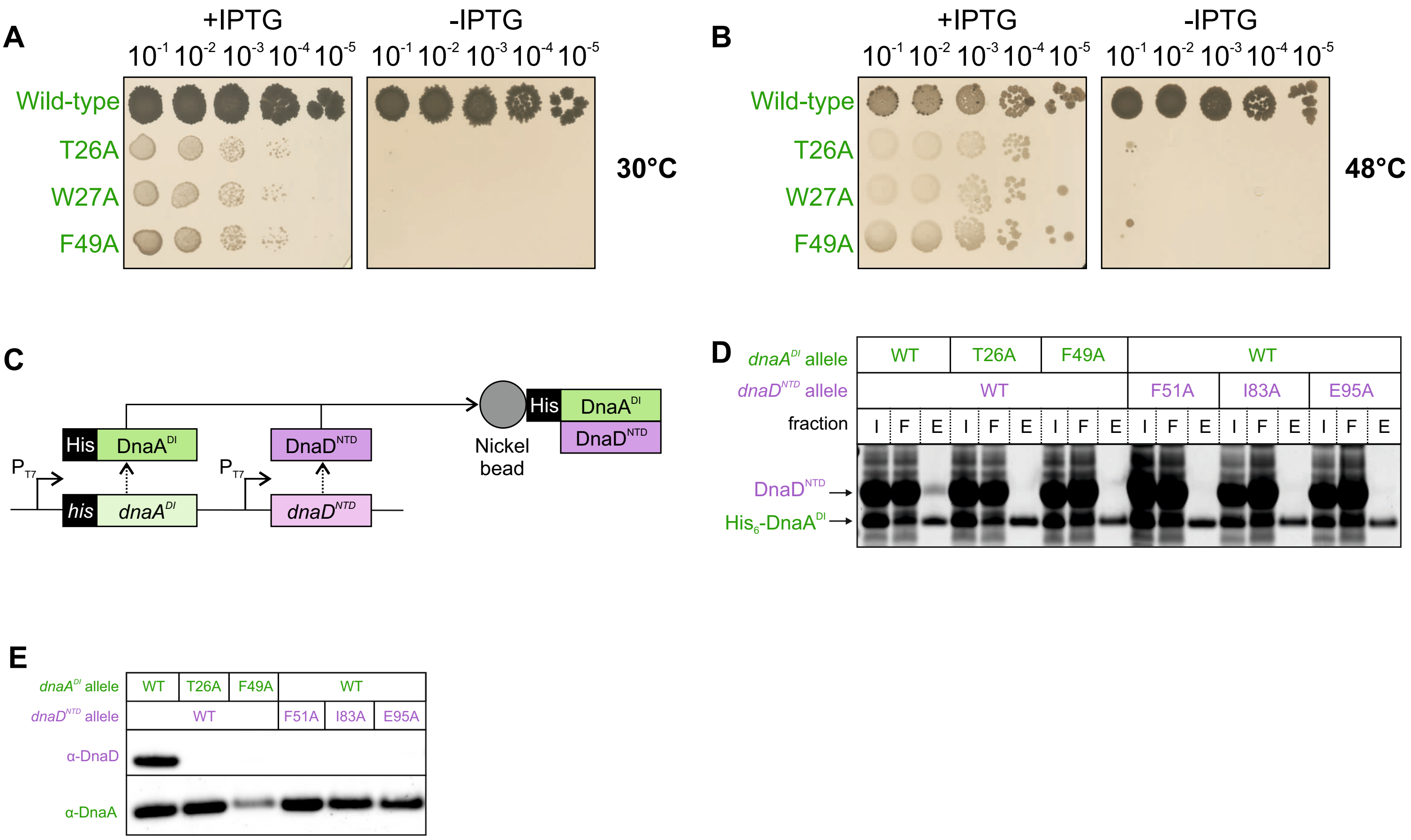

Figure S9. Residues in DnaA^{DI} disrupt the interaction between DnaA and DnaD. (A-B) Spot titre analyses showing that DnaA^{DI} variants produced a lethal phenotype *in vivo*. The presence or absence of IPTG indicates the induction state of the *repN/oriN* system. Wild-type (HM1108), T26A (HM1540), W27A (HM1541), F49A (HM1542). **(A)** shows DnaA domain I mutants lethal phenotype after 72h growth at 30°C. **(B)** shows DnaA domain I mutants lethal phenotype after 48h growth at 48°C. **(C)** Schematic of the pull-down assay using *his$_6$-dnaA^{DI}* and *dnaD^{NTD}* to probe for an interaction between DnaA domain I and the N-terminal domain of DnaD using Nickel beads. **(D)** Eluate staining from DnaA-DnaD pull-down assays showing loss of interaction between His$_6$-DnaA^{DI} and DnaD^{NTD} when using variants of DnaA or DnaD. WT indicates wild-type proteins and Input, Flow through and Eluate fractions are respectively indicated as I, F and E. **(E)** Immunoblot analysis of DnaA and DnaD mutant overexpression eluates from pull-down assays showing the identity of each polypeptide. Samples are the same as used in panel (D). Plasmids used in (D-E) are listed in Table S2.

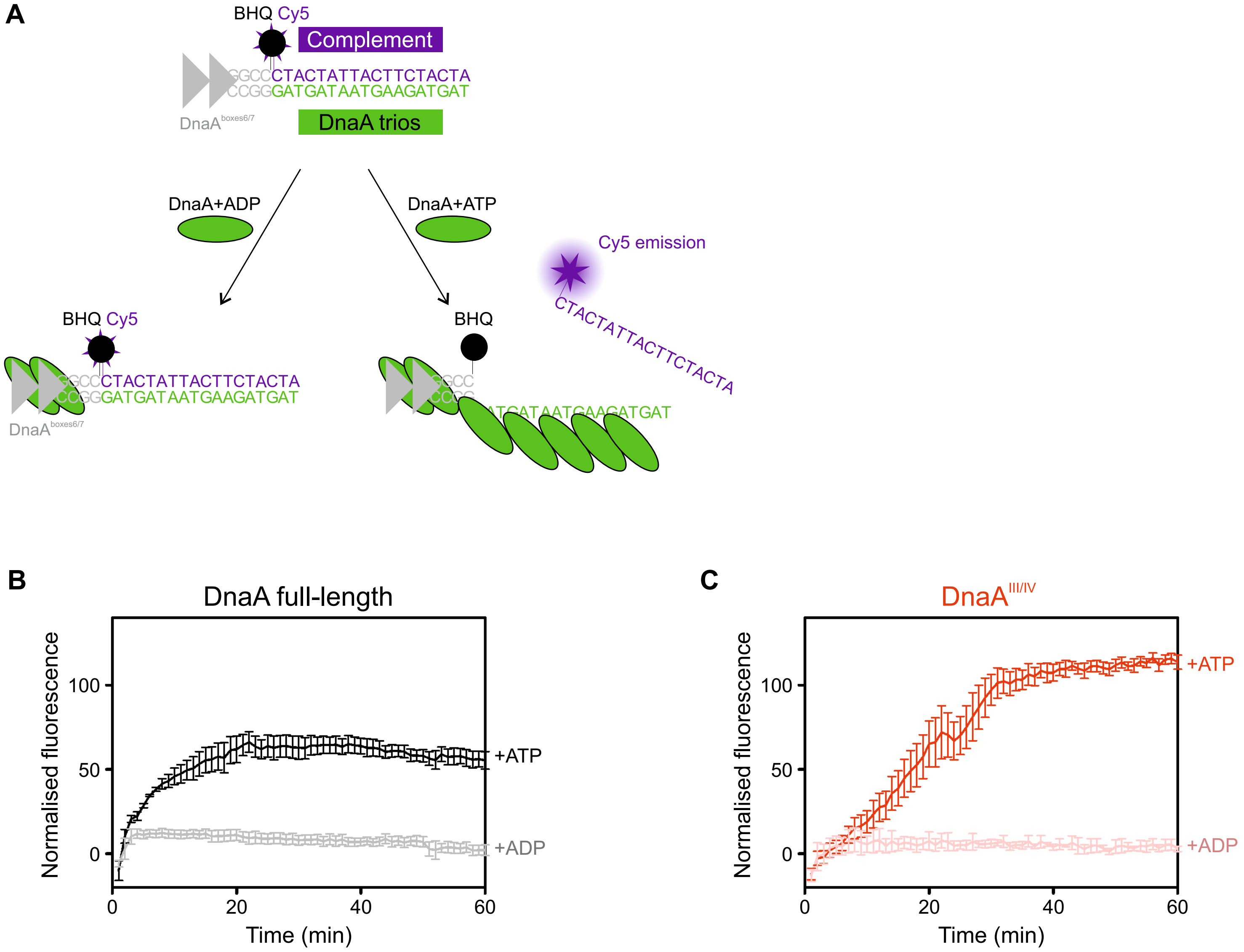

Figure S10. DnaA does not require domains I and II to unwind DNA substrates. (A) Illustration of the strand separation assay setup used to detect DnaA-directed unwinding of DNA substrates. Three oligonucleotides are annealed to mimick the *B. subtilis* origin unwinding region including DnaA boxes (dsDNA binding via DnaA) and the DnaA trios/complementary region. The bottom strand is continuous and unlabelled. The top strand corresponding to the DnaA boxes and GC-rich region is labelled with a black-hole quencher (BHQ) at the 3'-end and the complementary oligonucleotide to the trios is labelled with Cy5 at the 5'-end. As a fully dsDNA probe, the BHQ quenches fluorescence emitted by the Cy5 group. Upon incubation with DnaA and ADP, DnaA binds DnaA boxes, cannot engage the DnaA trios and no fluorescence remains quenched. In the presence of ATP, DnaA binds DnaA boxes and forms an oligomer on the DnaA trios, thereby displacing the probe complementary to the trios and allowing emission of Cy5 fluorescence. **(B-C)** Strand separation assays performed with the same probe in the presence of protein variants of DnaA. DNA substrate: oHM558/oHM778:oHM590. Background corresponding to the basal fluorescence of the DNA probe was subtracted from the curves. Error bars show the standard error of the mean for three biological replicates. **(B)** shows that DnaA full-length is able to separate strands in the presence of ATP. **(C)** shows that a truncation of DnaA lacking domains I and II retained the ability to separate strands in the presence of ATP.

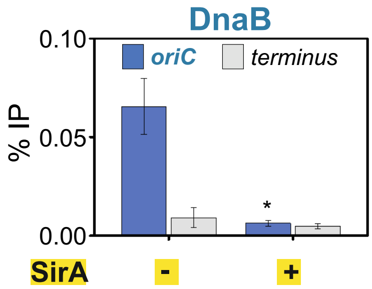

Figure S11. SirA overexpression abolishes DnaB recruitment to *oriC*. ChIP analysis showing that DnaB recruitment to *oriC* is lost following overexpression of SirA (HM1565). Primers used to amplify the origin annealed within the *incC* region. * shows a p-value of 0.0277.

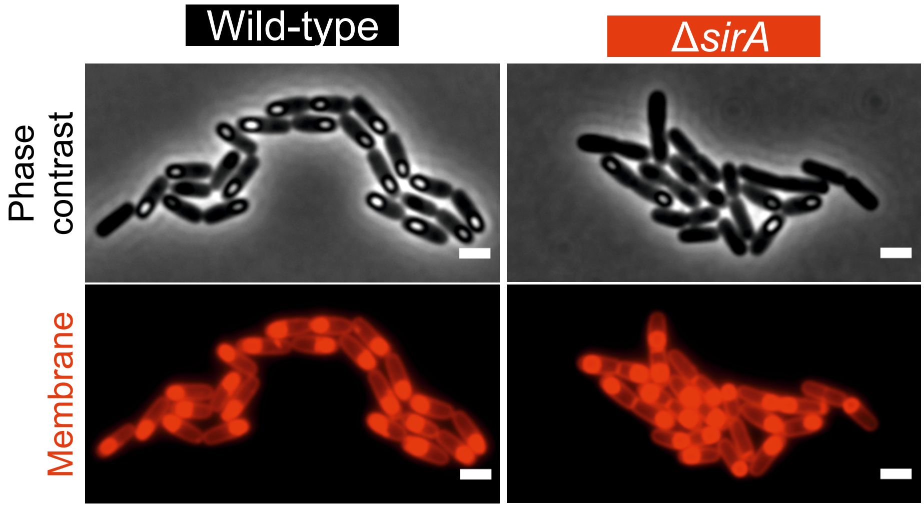

Figure S12. Wild-type and Δ*sirA* cells are both able to sporulate. Representative microscopy images showing that *B. subtilis* 168CA (wild-type) or cells lacking *sirA* (CW1065) were both able to sporulate by six hours after the induction of sporulation by the resuspension method. Phase bright entities show the formation of prespores in phase contrast images. Membrane staining via the Nile red dye highlights the location of spore formation within mother cells (bright red signal).

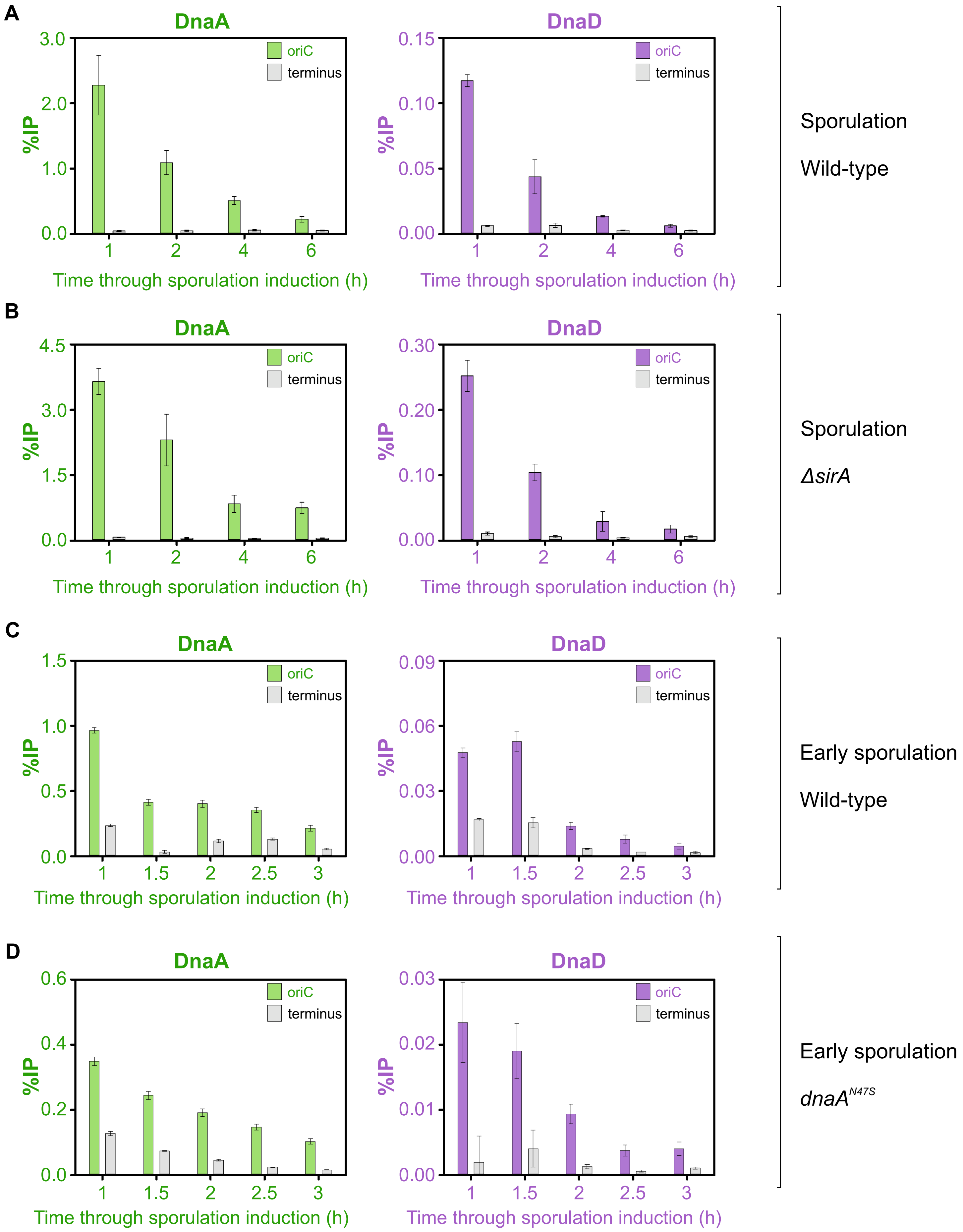

Figure S13. DnaA and DnaD are depleted from the origin during sporulation. (A-B) ChIP of DnaA and DnaD proteins at *oriC* throughout the induction of sporulation **(A)** in a wild-type strain (*B. subtilis* 168CA) and **(B)** in a knockout strain of *sirA* (CW1065). **(C-D)** ChIP of DnaA and DnaD proteins at *oriC* during early sporulation **(C)** in a wild-type strain (*B. subtilis* 168CA).and **(D)** in a strain suppressing the interaction between DnaA and SirA using the *dnaA*^{N47S} allele (CW1073). Primers used to amplify the *oriC* in (A-D) annealed within the *incC* region. Error bars show the standard error of the mean over three biological repeats.

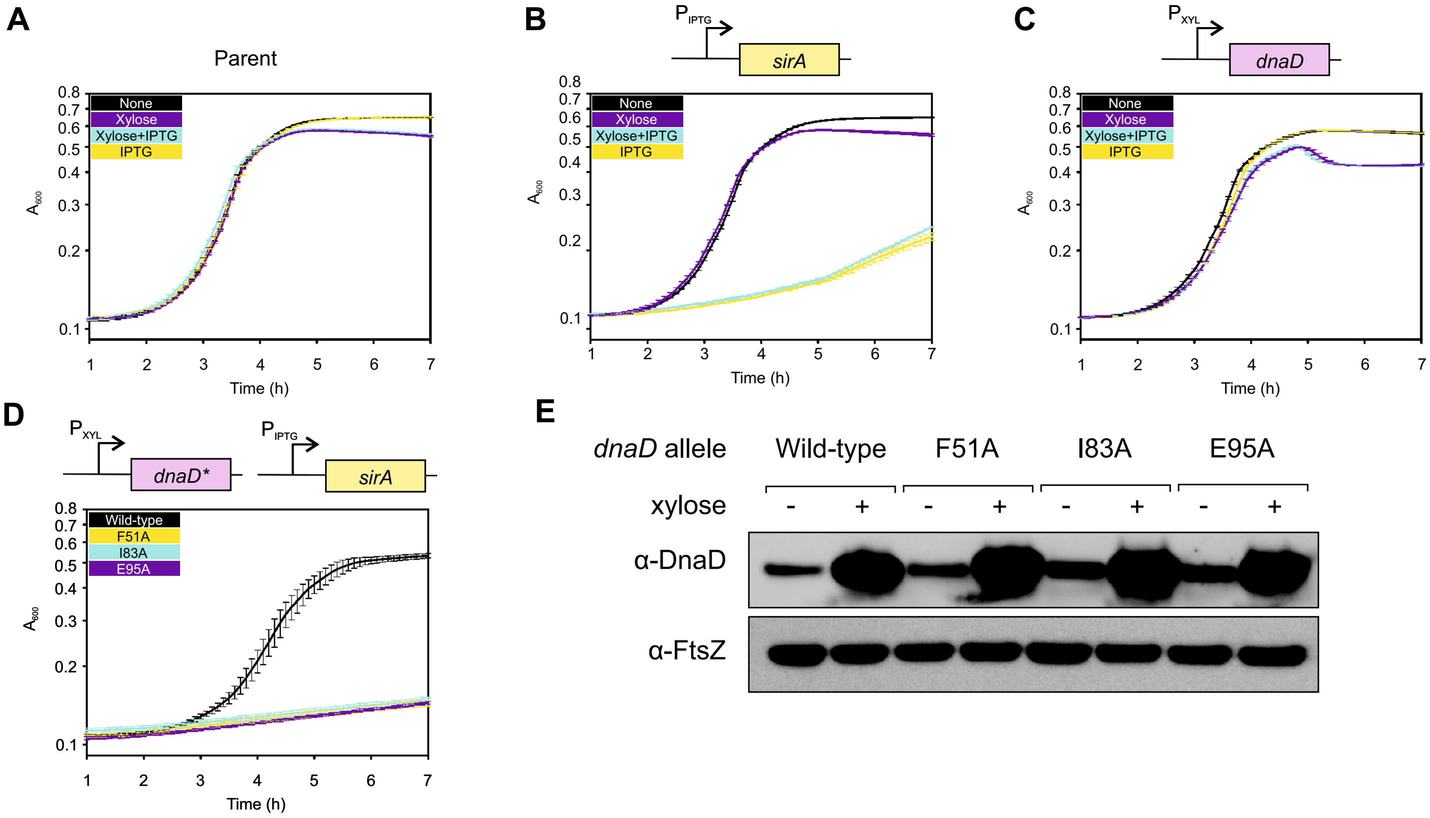

Figure S14. SirA inhibits the DnaA:DnaD interaction by preventing DnaD recruitment to *oriC*. (A-C) Plate reader growth assays with either no inducer (None), Xylose at 0.35%, IPTG at 0.035 mM or Xylose and IPTG together. **(A)** Shows that wild type *B. subtilis* 168CA grows in all conditions. **(B)** Shows that SirA overexpression in a strain background lacking the DnaD overexpression cassette inhibits bacterial growth, and that this inhibition is solely due to the addition of 0.035 mM IPTG (CW260). **(C)** Shows that DnaD overexpression in a strain background lacking the SirA overexpression cassette does not affect bacterial growth (CW261). **(D)** Plate reader analysis in the presence of xylose (0.35%) and IPTG (0.035 mM) shows that $DnaD^{NTD}$ mutants F51A, I83A and E95A do not rescue SirA-dependent growth inhibition. Wild-type (CW252), F51A (CW279), I83A (CW270), E95A (CW280). Error bars in (A-D) indicate the standard error of the mean for two biological replicates. **(E)** Immunoblot analysis showing that DnaD variants were overexpressed to similar levels following xylose induction (0.035%). Detection of the tubulin homolog FtsZ was used as a loading control. Strains are the same as used in (D).

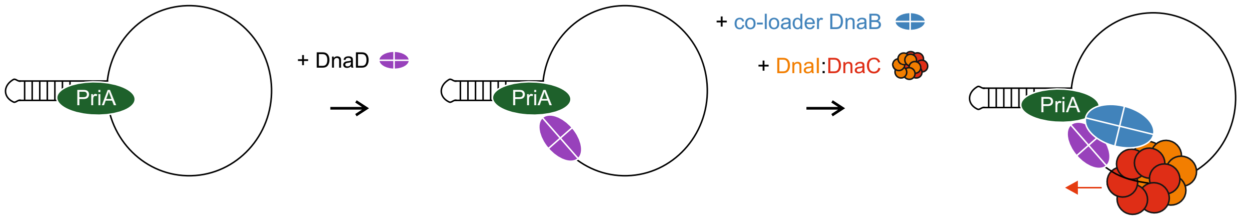

Figure S15. Model for helicase recruitment and loading in *B. subtilis* during PriA-dependent replication restart at a single-strand origin (*sso*).

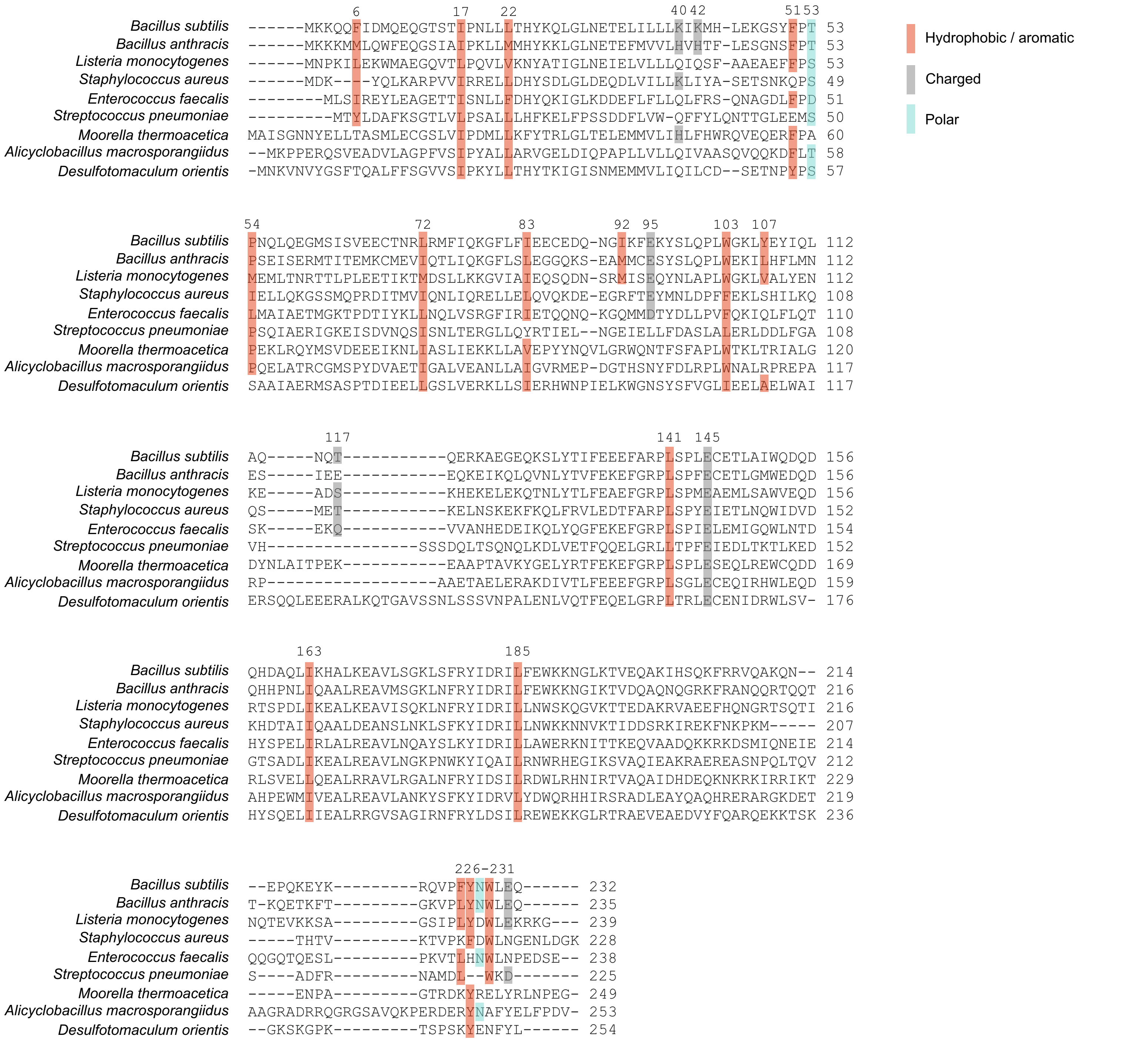

Figure S16. DnaD protein sequence conservation. Protein alignment showing the conservation of *B. subtilis* DnaD residues that displayed a phenotype *in vivo* and remained expressed. Numbers by the end of each row indicate amino-acid positions with respect to specific species and those on top of coloured boxes (red for hydrophobic / aromatic, grey for charged and blue for polar amino acid classes) are relative to the *B. subtilis* DnaD sequence.
